# Impact of the COVID-19 pandemic on antidepressant and antipsychotic use among children and adolescents: a population-based study

**DOI:** 10.3389/fped.2023.1282845

**Published:** 2023-12-11

**Authors:** Tony Antoniou, Kathleen Pajer, William Gardner, Melanie Penner, Yona Lunsky, Mina Tadrous, Muhammad Mamdani, Peter Gozdyra, David N. Juurlink, Tara Gomes

**Affiliations:** ^1^ Li Ka Shing Knowledge Institute, St. Michael’s Hospital, Toronto, ON, Canada; ^2^ ICES, Toronto, ON, Canada; ^3^ Department of Family and Community Medicine, University of Toronto, Toronto, ON, Canada; ^4^ Department of Family and Community Medicine, St. Michael’s Hospital, Toronto, ON, Canada; ^5^ Mental Health Program, Children’s Hospital of Eastern Ontario Research Institute, Ottawa, ON, Canada; ^6^ Department of Psychiatry, University of Ottawa, Ottawa, ON, Canada; ^7^ School of Epidemiology and Public Health, University of Ottawa, Ottawa, ON, Canada; ^8^ Autism Research Centre, Bloorview Research Institute, Holland Bloorview Kids Rehabilitation Hospital, Toronto, Canada; ^9^ Department of Pediatrics, University of Toronto, Toronto ON, Canada; ^10^ Azrieli Adult Neurodevelopmental Centre, Centre for Addiction and Mental Health, Toronto, Canada; ^11^ Department of Psychiatry, University of Toronto, Toronto, ON, Canada; ^12^ Leslie Dan Faculty of Pharmacy, University of Toronto, Toronto, ON, Canada; ^13^ Li Ka Shing Centre for Healthcare Analytics Research & Training, Unity Health Toronto, Toronto, ON, Canada; ^14^ Temerty Faculty of Medicine, University of Toronto, Toronto, ON, Canada; ^15^ Institute of Health Policy, Management, and Evaluation, University of Toronto, Toronto, ON, Canada; ^16^ Department of Medicine, University of Toronto, Toronto, ON, Canada

**Keywords:** antidepressant, antipsychotic agents, child, adolescent, time-series analysis, COVID-19 antidepressant, COVID-19

## Abstract

**Background:**

The COVID-19 pandemic was associated with increases in the prevalence of depression, anxiety and behavioural problems among children and youth. Less well understood is the influence of the pandemic on antidepressant and antipsychotic use among children. This is important, as it is possible that antidepressants and antipsychotics were used as a “stop-gap” measure to treat mental health symptoms when in-person access to outpatient care and school-based supportive services was disrupted. Furthermore, antipsychotics and antidepressants have been associated with harm in children and youth. We examined trends in dispensing of these medications two years following the pandemic among children 18 years of age and under in Ontario, Canada.

**Methods:**

We conducted a population-based time-series study of antidepressant and antipsychotic medication dispensing to children and adolescents ≤18 years old between September 1, 2014, and March 31, 2022. We measured monthly population-adjusted rates of antidepressant and antipsychotics obtained from the IQVIA Geographic Prescription Monitor (GPM) database. We used structural break analyses to identify the pandemic month(s) when changes in the dispensing of antidepressants and antipsychotics occurred. We used interrupted time series models to quantify changes in dispensing following the structural break and compare observed and expected use of these drugs.

**Results:**

Overall, we found higher-than-expected dispensing of antidepressants and antipsychotics in children and youth. Specifically, we observed an immediate step decrease in antidepressant dispensing associated with a structural break in April 2020 (−55.8 units per 1,000 individuals; 95% confidence intervals [CI] CI: −117.4 to 5.8), followed by an increased monthly trend in the rate of antidepressant dispensing of 13.0 units per 1,000 individuals (95% CI: 10.2–15.9). Antidepressant dispensing was consistently greater than predicted from September 2020 onward. Antipsychotic dispensing increased immediately following a June 2020 structural break (26.4 units per 1,000 individuals; 95% CI: 15.8–36.9) and did not change appreciably thereafter. Antipsychotic dispensing was higher than predicted at all time points from June 2020 onward.

**Conclusion:**

We found higher-than-expected dispensing of antidepressants and antipsychotics in children and youth. These increases were sustained through nearly two years of observation and are especially concerning in light of the potential for harm with the long-term use of antipsychotics in children. Further research is required to understand the clinical implications of these findings.

## Introduction

COVID-19-associated public health restrictions and school closures have been associated with a deterioration in children's and youth's mental health worldwide (1–3). A meta-analysis of 29 studies involving more than 80,000 children and youth found pooled prevalence estimates of depression and anxiety during the pandemic of 25.2% (95% CI, 21.2%–29.7%) and 20.5% (95% CI, 17.2%–24.4%), respectively (4). Similar findings were observed in other systematic reviews comprising predominantly cross-sectional studies (5–7). These findings have been extended by a meta-analysis of 21 longitudinal studies of more than 96,000 children and youth, with increases in depression, anxiety, distress, loneliness and negative affect reported during the pandemic relative to pre-pandemic periods (8). Behavioural problems were also common during the pandemic, with one meta-analysis of 66 studies finding a prevalence of 27% (95% CI: 19%–36%) in high-income countries (9). In addition, findings from an international online survey found that home confinement during COVID was associated with negative effects on mental wellbeing and emotional status, as well as physical and social inactivity (10). The pandemic has also been associated with changes in the use of mental health services, with population-based studies from Canada reporting increased outpatient mental health service utilization between July 2020 and February 2021 and higher than expected mental health visit rates in pediatric hospitals during most of the pandemic (11, 12).

Despite these studies, comparatively little research has evaluated pandemic-associated changes in the prescribing of psychotropic drugs among children and youth (13–17). Research into antidepressant and antipsychotic use is especially important for several reasons. First, few of these drugs have regulatory approval for use in children, with antipsychotics being primarily used off-label for managing non-psychotic disorders and externalizing symptoms in children and youth (18, 19). In addition, it is possible that antidepressants and antipsychotics were used as a “stop-gap” measure to treat mental health symptoms when in-person access to non-pharmacologic supportive services was disrupted. Furthermore, sustained use following the return to in-person care may suggest persistent mental health symptoms and/or difficulties weaning these medications. Finally, antipsychotics can cause metabolic disturbances and weight gain, increasing the risk of type 2 diabetes, cardiovascular disease, and sudden death, while antidepressants have been associated with an increased risk of cardiac events and suicidal thoughts (20–23).

In light of the lack of research examining long-term trends in the use of antipsychotics and antidepressants in children and youth and potential concerns regarding sustained increased use post-pandemic, our study objective was to examine changes in the dispensing of these medications to children 18 years of age and under in Ontario, home to approximately 40% of Canadian children (24).

## Methods

### Setting and study design

We conducted a population-based study of antidepressant and antipsychotic units dispensed monthly by Ontario community pharmacies to children, regardless of payer, between September 1, 2014, and March 31, 2022. As in other jurisdictions, Ontario children and youth experienced several cycles of school openings and closures during the pandemic, beginning in March 2020, with all students learning remotely until June 2020. In September 2020, students were given the choice of in-person or remote leaning until January 2021, at which time most students returned to remote learning until February 2021. In April 2021, schools were closed province-wide for the remainder of the school year. Schools re-opened in September 2021 with the option of remote learning. A final brief period of school closures and remote learning was implemented in January 2022 (see Supplementary appendix for timeline) (25). Overall, Ontario students experienced the most frequent fully-remote school closures in Canada, totalling approximately 220 days during the pandemic (26).

### Data sources

We used the IQVIA Geographic Prescription Monitor (GPM) database. The IQVIA GPM database provides projections of prescription drug utilization in all Canadian provinces based on the IQVIA retail prescription database, using a representative sample of more than 6,100 pharmacies across Canada. The patented geospatial projection methodology is used to generate monthly prescription estimates that are representative of drug utilization at the provincial level (27). These data are monitored and validated by IQVIA and are routinely used for international research on drug utilization trends (28–32). This data source includes aggregate prescription units from public and private payers. We used Statistics Canada's annual population estimates for Ontario to population-adjust dispensing rates (33). Because of limitations in data availability, we could not study young adults aged 19 to 24, and restricted our analyses to children and adolescents aged 18 years and younger. Furthermore, the IQVIA database does not provide additional patient-level data, precluding analysis on the number of children and youth affected or an examination of trends by other variables, such as sex, race or ethnicity.

### Ethics

Research ethics approval was not required for this study because the researchers had access only to aggregated, de-identified data.

### Outcomes

Our primary outcome was the monthly population-adjusted rate of medication units (i.e., tablets, capsules or prefilled syringes of long-acting formulations of antipsychotics) dispensed per 1,000 children, stratified by drug type (i.e., antidepressants or antipsychotics) and age category (≤13 years and 14 to 18 years). We included long-acting injectable dosage forms of antipsychotics because they are commonly dispensed as prefilled syringes or set dose packs that can be converted to units. We excluded short-acting injectable dosage forms of antipsychotics because they are not commonly used in this population and because precise units of short-acting injectable doses are difficult to quantify.

### Statistical analysis

We used several approaches to explore whether antidepressant and antipsychotic dispensing to children and youth changed during the pandemic. We began by first testing whether there were changes in the antipsychotic and antidepressant dispensing rate during the study period and identifying the date(s) of these changes. We next proceeded to quantify the changes in dispensing following these date(s). Finally, we compared observed dispensing rates with those that would have been predicted in the absence of COVID-19. Specifically, we first used structural break analyses to test for changes in antidepressant and antipsychotic dispensing during the pandemic, following seasonal adjustment and 15% trimming of the dataset (34). Next, we used interrupted time series analyses to quantify the change in monthly dispensing trends following the structural break (35, 36). Specifically, we used a dummy variable to denote the timing of the structural break(s), an indicator for time to account for the underlying temporal trend in the data, and an interaction term between time and the dummy variable representing the structural break to estimate the change in dispensing temporal trend following the structural break. Our models also included dummy variables indicating the month to account for seasonality and a variable denoting the implementation of a publicly funded pharmacare program known as OHIP+ that covered the prescription costs of all individuals aged 24 and under, beginning in January 2018 (37). We also determined expected antidepressant and antipsychotic dispensing rates for the period following the structural break in the absence of COVID-19 using data from January 2013 to the month preceding the structural break, with pre-structural break time, month, and the indicator for OHIP+ as model predictors. We then determined the relative percent differences between the observed and predicted stimulant dispensing rates and estimated associated 95% confidence intervals using the Poisson distribution. We tested models for autocorrelation to a maximum of 12 lags using the Cumby-Huizinga test for autocorrelation and estimated all models using Newey-West standard errors to account for autocorrelation up to 12 lags and heteroskedasticity (38, 39). All analyses used Stata version 17.0 (StataCorp LLC, College Station, TX, USA) and EViews 12.

## Results

### Summary

Overall, we found higher than expected dispensing of antidepressants and antipsychotics in children and youth during the pandemic. Specifically, we observed an immediate decrease in antidepressant dispensing in April 2020, followed by an increased monthly trend in the rate of antidepressant dispensing of 13.0 units per 1,000 individuals. Antidepressant dispensing was consistently greater than predicted from September 2020 onward. Antipsychotic dispensing increased immediately following June 2020, did not change appreciably thereafter, and was higher than predicted at all time points from June 2020 onward.

### Antidepressant dispensing following COVID-19

Structural break analyses identified breaks in the endogenous series occurring in March 2018, approximating the month of OHIP+ implementation, and April 2020, the month following the declaration of a public health emergency and school closures. In descriptive analyses, we observed a decrease in antidepressant dispensing to children and youth immediately following the April 2020 structural break, with a relative percent decrease in rates of 21.1% (95% CI: −21.3% to −21.0%) between March 2020 and May 2020 (883.8 vs. 697.0 units per 1,000 population) (Table 1). However, antidepressant use increased in the months following the April 2020 structural break, with a relative percent increase of 21.4% (95% CI 21.2%–21.6%) between March 2020 and March 2022 (883.8 vs. 1,072.6 units per 1,000 population (Table 1).

**Table 1:** Changes in antidepressant and antipsychotic dispensing following COVID-19 pandemic.

	COVID Structural Break(s)	Rates and relative percent changes					Interrupted time series	
	Structural break(s)	Rate per 1,000, in month preceding break	Rate per 1,000 in month following break	Relative percent change (95% CI), month preceding break to month after	Rate per 1,000 March 2022	Relative per cent change, month preceding break to March 2022	Change in dispensing rate per 1,000, first month following break	Monthly change in dispensing rate trend per 1,000 post-break
Antidepressants								
Overall	April 2020	883.8	697.0	−21.1% (−21.3% to −21.0%)	1,072.6	21.4% (21.2% to 21.6%)	−55.8 (−117.4 to 5.8)	13.0 (10.2 to 15.9)
Age								
0–13	April 2020	274.8	220.7	−19.7% (−20.0% to −19.4%)	322.3	17.3% (16.9% to 17.7%)	−9.8 (−31.0 to 11.4)	3.8 (2.7 to 4.8)
14–18	April 2020	2,467.8	1,935.9	−21.6% (−21.7% to −21.4%)	3,002.6	21.7% (21.4% to 21.9%)	−168.8 (−340.7 to 3.2)	37.0 (28.7 to 45.3)
Antipsychotics								
Overall	June 2020	253.3	299.1	18.1% (17.7% to 18.4%)	286.7	13.2% (12.8% to 13.5%)	26.4 (15.8 to 36.9)	0.35 (−0.26 to 0.96)
Age								
0–13	June 2020	148.7	183.5	23.4% (22.8% to 24.0%)	165.6	11.3% (10.8% to 11.9%)	20.9 (13.6 to 28.2)	−0.37 (−0.73 to 0.00)
14–18	June 2020	525.3	599.7	14.2% (13.7% to 14.6%)	598.3	13.9% (13.4% to 14.4%)	41.6 (20.0 to 63.1)	2.2 (0.45 to 3.9)

**Figure 1:**
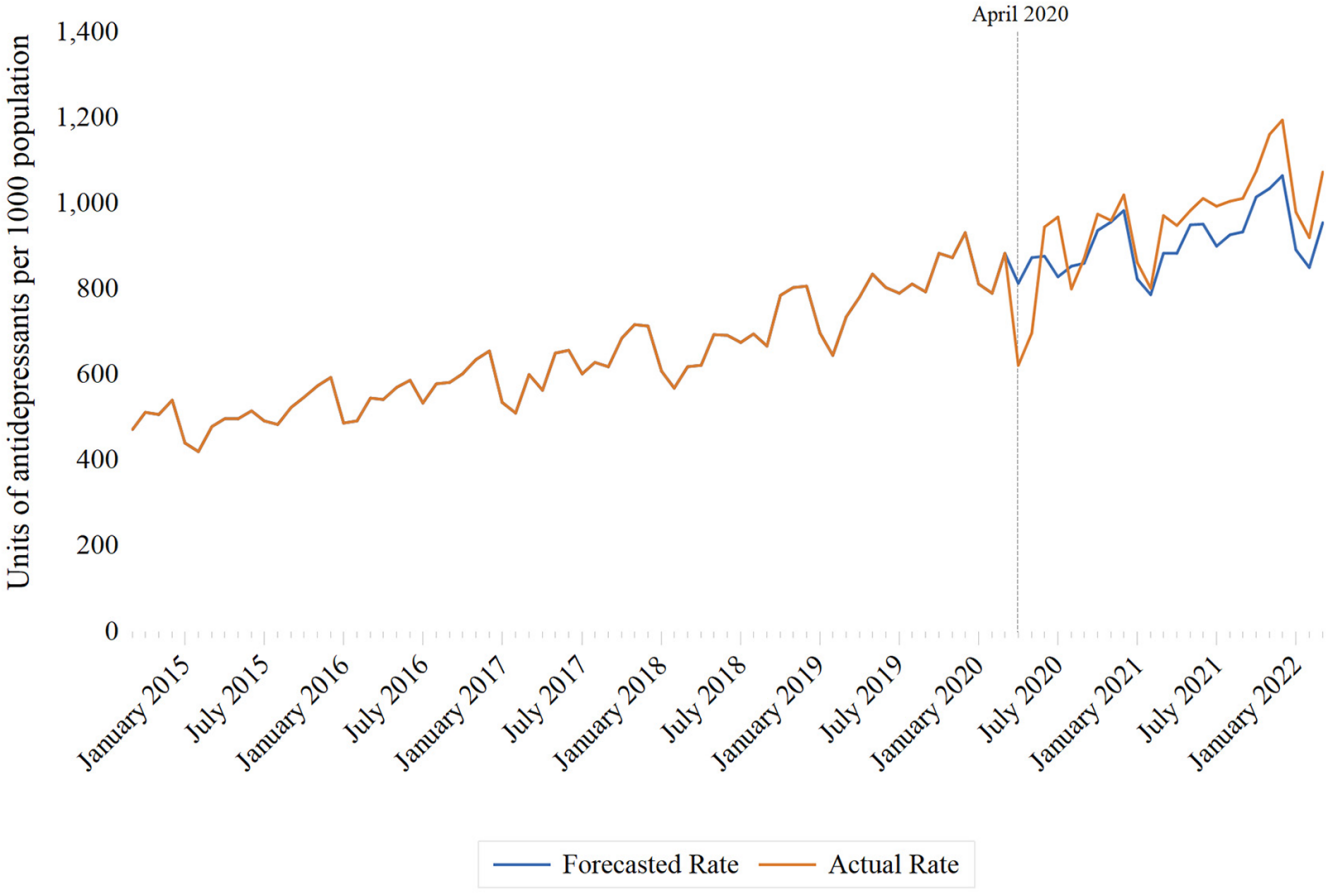
Monthly rates of antidepressant dispensing per 1,000 Ontario residents aged 18 years and under, September 2014 to March 2022.

Following interrupted time series modelling, there was an immediate decline in antidepressant dispensing of 55.8 units per 1,000 individuals (95% CI: −117.4 to 5.8), with a larger decrease observed among adolescents between the ages of 14 and 18 (−168.8 units per 1,000 individuals; 95% CI: −340.7 to 3.2) relative to children aged 0–13 years (−9.8 units per 1,000 individuals; 95% CI: −31.0 to 11.4) (Table 1). These findings corresponded to lower than forecasted dispensing rates in April 2020 (−23.5%; 95% CI: −23.6% to −23.3%) and May 2020 (−20.2% (95% CI: −20.3% to −20.0%) (Supplementary Table S1; Figure 1). Following the initial decline, there was an increased trend in the monthly rate of antidepressant dispensing of 13.0 units per 1,000 individuals (95% CI: 10.2–15.9) (Table 1). The increased trend was greater among adolescents aged 14–18 (37.0 units per 1,000 individuals; 95% CI: 28.7–45.3) than children between the ages of 0 and 13 (3.8 units per 1,000 individuals; 95% CI: 2.7–4.8) (Table 1, Supplementary Figure S2).

In analyses comparing observed and forecasted rates, higher than expected antidepressant rates were first observed in June 2020, with a relative percent difference of 7.8% (95% CI: 7.7%–8.0%). Observed antidepressant rates were consistently higher than predicted rates from predicted rates from September 2020 onward (Supplementary Tables S1–S3).

### Antipsychotic dispensing following COVID-19

Structural breaks in the antipsychotic dispensing time series were also observed in March 2018 (aligned with OHIP+) as well as in June 2020. We observed an 18.1% (95% CI: 17.7%–18.4%) relative percent increase in antipsychotic dispensing between May and July 2020 (253.3 vs. 299.1 units per 1,000 individuals, respectively) (Table 1). Antipsychotic dispensing remained elevated following the June 2020 structural break, with a relative percent increase of 13.2% (95% CI: 12.8%–13.5%) between March 2020 and March 2022 (253.3 vs. 286.7 units per 1,000 individuals).

Interrupted time series models estimated an immediate step increase in antipsychotic dispensing of 26.4 units per 1,000 individuals (95% CI: 15.8–36.9) following the June 2020 structural break (Table 1). The initial increase was greater among adolescents aged 14 to 18 (41.6 units per 1,000 individuals; 95% CI: 20.0–63.1) relative to children between the ages of 0 and 13 (20.9 units per 1,000 individuals; 95% CI: 13.6–28.2) (Supplementary Figure S2). The monthly antipsychotic dispensing trend did not change appreciably following the initial increase (0.35 units per 1,000 individuals; 95% CI: −0.26 to 0.96), although a sustained increasing monthly trend was observed among adolescents between the ages of 14 and 18 years (2.2 units per 1,000 individuals; 95% CI: 0.45–3.9) in stratified analyses (Table 1).

**Figure 2:**
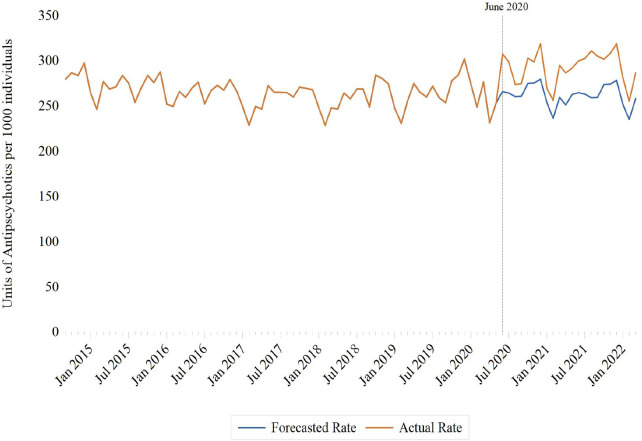
Monthly rates of antipsychotic dispensing per 1,000 Ontario residents aged 18 years and under, September 2014 to March 2022.

In analyses comparing observed and forecasted rates, antipsychotic dispensing was higher than predicted at all time points from June 2020 onward, overall and in analyses stratified by age (Figure 2; Supplementary Tables S4–S6).

## Discussion

In our study, we found sustained post-pandemic increases in pediatric antidepressant and antipsychotic dispensing, particularly among adolescents between the ages of 14 and 18. Although antidepressant use initially declined, dispensing rates consistently exceeded predicted rates from September 2020 onward. In contrast, antipsychotic dispensing rates increased immediately beginning in June 2020 and remained above predicted rates at all time points thereafter. The timing of these changes corresponds with the onset of the second pandemic wave in Ontario, Canada. This period was notable for multiple cycles of school closures and re-openings and the implementation of masking and physical distancing in schools, measures which limited opportunities for socialization and the social learning activities conducive to supporting mental health (40). Although the observational nature of our study limits our ability to infer causality, the use of structural break analyses and interrupted time series models supports a temporal relationship between the COVID-19 pandemic and increased use of antidepressants and antipsychotics in children and youth.

Our findings of increased antidepressant and antipsychotic dispensing complement those of an earlier population-based study of mental health-related health service use among Ontario children and youth aged 3–17. Specifically, this study found a 10%–15% increase in expected outpatient visits for mood and anxiety disorders and a 9% to 30% increase in visits for psychotic disorders beginning in July 2020 (11), corresponding roughly to our findings of increased antidepressant and antipsychotic dispensing beginning around June 2020. The initial decline in antidepressant dispensing coincides with lower-than-expected rates of mental health outpatient visits among Ontario children and youth during the first two months of the pandemic, with a nadir observed in April 2020 (11, 41). Furthermore, assessments by mental health agencies caring for children and youth declined 50% during the first two months of the pandemic, limiting access and referrals to providers (42). Alternatively, it is possible that school closures were initially associated with improved mental health in some children and youth experiencing school-related social and performance anxieties (43, 44). Our results are also similar to those from other jurisdictions associating the pandemic with an increased prevalence of mental health symptoms and greater use of antidepressants and antipsychotics among children and youth (4–9). Our work extends these findings by demonstrating that increased use of antidepressants and antipsychotics observed early in the pandemic persisted even as some public health measures were relaxed.

Although our data do not allow us to determine reasons for persistently elevated antidepressant and antipsychotic use, one possibility is that pandemic-associated stress and increased experience with family stressors have resulted in a chronic deterioration in the mental health of children and youth (42). This assertion is supported by research demonstrating increases in depressive symptoms and self-injury among Ontario children and youth between the first and second waves of the pandemic and international research showing an increased prevalence of anxiety, depression and sleep disorders through October 2022 (42, 45). The use of antidepressants for the treatment of persistent COVID-related mental health symptoms may have also contributed to increased use of these drugs (46). Further longitudinal research is required to understand the long-term impact of COVID-19 on the mental health of children and youth (47). Another contributing factor to persistently high psychotropic use is difficulty weaning medication during the pandemic, mainly during the period when most care was delivered virtually. This is especially problematic considering the potential for harm with antipsychotics. Specifically, these drugs have been associated with dyslipidemia, obesity and type 2 diabetes, the risks of which increase with cumulative dose and treatment duration (48–50). Because antipsychotic use did not return to pre-pandemic levels following an initial increase in use, our findings suggest that the number of children at risk of antipsychotic-induced metabolic disorders has increased during the pandemic. Moreover, the transition to virtual care likely exacerbated pre-pandemic findings of low adherence to metabolic monitoring in antipsychotic-treated children and youth, further increasing the risk of harm (51, 52). Further research is necessary to clarify the clinical impacts and potential for harm with the increased antipsychotic use among children and youth.

Our study has some limitations. First, we could not assess the appropriateness of, or indications for, antidepressant or antipsychotic medication dispensing. Prior Canadian research has found high levels of off-label antipsychotic use in children and youth, with most prescriptions being written to address disruptive behavior disorders, depression, and anxiety (53). Second, we were limited by the prescription-level data, which did not allow us to examine individual-level characteristics associated with dispensing trends. We are therefore unable to comment on whether pandemic-associated changes in dispensing differed among various strata of children and youth. We are also unable to determine if certain demographic groups or regions are underrepresented in the data. This may be especially important for low-income children and youth, who our prior research demonstrated were more likely to receive antipsychotics than those from higher-income families (54). Furthermore, findings of disproportionate increases in antipsychotic use among specific sub-populations of children and youth could have ethical implications if related to differential access to non-pharmacologic treatments. Third, our study was conducted in a single Canadian province, potentially limiting the generalizability of our findings. However, our findings generally align with those of others examining pandemic-associated changes in psychotropic drug use in children and youth (14–17). Moreover, we are unable to generalize our findings to young adults between the ages of 19–24. Fourth, we were unable to distinguish trends among drugs with different potential for metabolic adverse effects in children and youth (55). Finally, our study period comprised the first 24 months of the pandemic. Additional longitudinal research is required to understand the post-pandemic evolution in the use of psychotropic medications in children and youth.

## Conclusion

We found higher-than-expected dispensing of antidepressants and antipsychotics in children and youth. These increases were sustained through nearly two years of observation and are especially concerning in light of the potential for harm with the long-term use of antipsychotics in children. Further research is required to understand the clinical implications of these findings, examine whether changes in post-pandemic use of antipsychotics and antidepressants varied among different sub-populations of children and youth, whether such differences reflect differential access to non-pharmacologic therapies, and develop strategies supporting the uninterrupted provision of non-pharmacologic therapies to children and youth with mental health conditions during future pandemics and other periods of long-term stressors.

## Data Availability

The statements, findings, conclusions, views, and opinions expressed in this study are based in part on data obtained under license from IQVIA Solutions Canada Inc. The statements, findings, conclusions, views, and opinions expressed herein are not necessarily those of IQVIA Inc. or any of its affiliated or subsidiary entities. The data that support the findings of this study are available from IQVIA www.iqvia.com). Restrictions apply to the availability of these data, which were used under license for this study.

## References

[1] Cama SF Miyamoto BE DeJong SM. Impact on child psychiatry. Psychiatr Clin North Am. (2022) 45:133–46. 10.1016/j.psc.2021.11.009 35219434 PMC9756428

[2] Rogers AA Ha T Ockey S. Adolescents’ perceived socio-emotional impact of COVID-19 and implications for mental health: results from a U.S.-based mixed-methods study. J Adolesc Health. (2021) 68:43–52. 10.1016/j.jadohealth.2020.09.039 33143986 PMC7605752

[3] Cost KT Crosbie J Anagnostou E Birken CS Charach A Monga S Mostly worse, occasionally better: impact of COVID-19 pandemic on the mental health of Canadian children and adolescents. Eur Child Adolesc Psychiatry. (2022) 31:671–84. 10.1007/s00787-021-01744-3 33638005 PMC7909377

[4] Racine N McArthur BA Cooke JE Eirich R Zhu J Madigan S. Global prevalence of depressive and anxiety symptoms in children and adolescents during COVID-19: a meta-analysis. JAMA Pediatr. (2021) 175:1142–50. 10.1001/jamapediatrics.2021.2482 34369987 PMC8353576

[5] Ma L Mazidi M Li K Li Y Chen S Kirwan R Prevalence of mental health problems among children and adolescents during the COVID-19 pandemic: a systematic review and meta-analysis. J Affect Disord. (2021) 293:78–89. 10.1016/j.jad.2021.06.021 34174475 PMC9711885

[6] Nearchou F Flinn C Niland R Subramaniam SS Hennessy E. Exploring the impact of COVID-19 on mental health outcomes in children and adolescents: a systematic review. Int J Environ Res Public Health. (2020) 17:8479. 10.3390/ijerph17228479 33207689 PMC7698263

[7] Panchal U Salazar de Pablo G Franco M Moreno C Parellada M Arango C The impact of COVID-19 lockdown on child and adolescent mental health: systematic review. Eur Child Adolesc Psychiatry. (2023) 32:1151–77. 10.1007/s00787-021-01856-w 34406494 PMC8371430

[8] Kauhanen L Wan Mohd Yunus WMA Lempinen L Peltonen K Gyllenberg D Mishina K A systematic review of the mental health changes of children and young people before and during the COVID-19 pandemic. Eur Child Adolesc Psychiatry. (2023) 32:995–1013. 10.1007/s00787-022-02060-0 35962147 PMC9373888

[9] Peng B Reeves KKL Lee SWY Chung THY Hui HWL Leung AHL Physical, psychological, and behavioral problems among children and adolescents in countries with different economic statuses during the COVID-19 pandemic: a systematic review and meta-analysis. Front Pediatr. (2023) 11:1181186. 10.3389/fped.2023.1181186 37342536 PMC10277820

[10] Ammar A Trabelsi K Brach M Chtourou H Boukhris O Masmoudi L Effects of home confinement on mental health and lifestyle behaviours during the COVID-19 outbreak: insights from the ECLB-COVID19 multicentre study. Biol Sport. (2021) 38:9–21. 10.5114/biolsport.2020.96857 33795912 PMC7996377

[11] Saunders NR Kurdyak P Stukel TA Strauss R Fu L Guan J Utilization of physician-based mental health care services among children and adolescents before and during the COVID-19 pandemic in Ontario, Canada. JAMA Pediatr. (2022) 176:e216298. 10.1001/jamapediatrics.2021.6298 35129604 PMC8822447

[12] Saunders NR Stukel TA Strauss R Fu L Cohen E Guttmann A Changes in hospital-based care seeking for acute mental health concerns among children and adolescents during the COVID-19 pandemic in Ontario, Canada, through September 2021. JAMA Netw Open. (2022) 5:e2220553. 10.1001/jamanetworkopen.2022.20553 35797049 PMC9264033

[13] Bliddal M Rasmussen L Andersen JH Jensen PB Pottegård A Munk-Olsen T Psychotropic medication use and psychiatric disorders during the COVID-19 pandemic among Danish children, adolescents, and young adults. JAMA Psychiatry. (2023) 80:176–80. 10.1001/jamapsychiatry.2022.4165 36515919 PMC9856810

[14] Wood SJ Ilomäki J Gould J Tan GS Raven M Jureidini JN Dispensing of psychotropic medications to Australian children and adolescents before and during the COVID-19 pandemic, 2013-2021: a retrospective cohort study. Med J Aust. (2023) 219(1):18–25. 10.5694/mja2.51948 37182907

[15] Amill-Rosario A Lee H Zhang C dosReis S. Psychotropic prescriptions during the COVID-19 pandemic among U.S. children and adolescents receiving mental health services. J Child Adolesc Psychopharmacol. (2022) 32:408–14. 10.1089/cap.2022.0037 36067121

[16] Zandy M El Kurdi S Samji H McKee G Gustafson R Smolina K. Mental health-related healthcare service utilisation and psychotropic drug dispensation trends in British Columbia during COVID-19 pandemic: a population-based study. Gen Psychiatr. (2023) 36:e100941. 10.1136/gpsych-2022-100941 36875149 PMC9971830

[17] Leong C Katz LY Bolton JM Enns MW Delaney J Tan Q Psychotropic drug use in children and adolescents before and during the COVID-19 pandemic. JAMA Pediatr. (2022) 176:318–20. 10.1001/jamapediatrics.2021.5634 34982101 PMC8728654

[18] Sohn M Moga DC Blumenschein K Talbert J. National trends in off-label use of atypical antipsychotics in children and adolescents in the United States. Medicine (Baltimore). (2016) 95:e3784. 10.1097/MD.0000000000003784 27281081 PMC4907659

[19] Hoekstra PJ Dietrich A. First do no harm: use off-label antipsychotic medication in children and adolescents with great caution. Eur Child Adolesc Psychiatry. (2022) 31:1–3. 10.1007/s00787-022-01950-7 35064827

[20] Correll CU Manu P Olshanskiy V Napolitano B Kane JM Malhotra AK. Cardiometabolic risk of second-generation antipsychotic medications during first-time use in children and adolescents. JAMA. (2009) 302:1765–73. 10.1001/jama.2009.1549 19861668 PMC3055794

[21] Rubin DM Kreider AR Matone M Huang YS Feudtner C Ross ME Risk for incident diabetes mellitus following initiation of second-generation antipsychotics among medicaid-enrolled youths. JAMA Pediatr. (2015) 169:e150285. 10.1001/jamapediatrics.2015.0285 25844991

[22] Ray WA Stein CM Murray KT Fuchs DC Patrick SW Daugherty J Association of antipsychotic treatment with risk of unexpected death among children and youths. JAMA Psychiatry. (2019) 76:162–71. 10.1001/jamapsychiatry.2018.3421 30540347 PMC6440238

[23] Dwyer JB Bloch MH. Antidepressants for pediatric patients. Curr Psychiatr. (2019) 18:26–42F. 31511767 PMC6738970

[24] Statistics Canada. Focus on geography series, 2016 census. Available at: https://www12.statcan.gc.ca/census-recensement/2016/as-sa/fogs-spg/Index-eng.cfm (Accessed June 24, 2023).

[25] Canadian Institute for Health Information. Canadian COVID-19 intervention timeline—data tables. Ottawa, ON: CIHI (2022).

[26] Han JY Breton C. Have provinces put students first during COVID? Available at: https://policyoptions.irpp.org/magazines/february-2022/have-provinces-put-schools-first-during-covid/ (Accessed August 14, 2023).

[27] Boardman C, inventor. *IMS Health incorporated, assignee. System and method for estimating prdouct distribution using a product specific universe*. US patent 7,174,304. (2007).

[28] Blais JE Wei Y Yap KKW Alwafi H Ma TT Brauer R Trends in lipid-modifying agent use in 83 countries. Atherosclerosis. (2021) 328:44–51. 10.1016/j.atherosclerosis.2021.05.016 34091069

[29] Gunning R Chu C Nakhla N Kim KC Suda KJ Tadrous M. Major shifts in acid suppression drug utilization after the 2019 ranitidine recalls in Canada and United States. Dig Dis Sci. (2023) 68:3259–67. 10.1007/s10620-023-07958-6 37269368 PMC10238237

[30] Ben Hassen C Tahir R Singh-Manoux A Milic D Paquet C Sabia S Ten-tear trends in sales of Alzheimer disease drugs in France compared with sales in Germany, Spain, and the UK. JAMA Health Forum. (2022) 3:e222253. 10.1001/jamahealthforum.2022.2253 36003418 PMC9356313

[31] Black CD McCarthy L Gomes T Mamdani M Juurlink D Tadrous M. Interprovincial variation of psychotropic prescriptions dispensed to older Canadian adults. Can Geriatr J. (2018) 21:269–73. 10.5770/cgj.21.307 30271512 PMC6136906

[32] Ontario Drug Policy Research Network. Antipsychotic use in the elderly—final consolidated report. Toronto, ON: The Network (2015).

[33] Statistics Canada. Annual demographic estimates: Canada, provinces and territories Ottawa, ON2021. Available at: https://www150.statcan.gc.ca/n1/en/catalogue/91-215-X (Accessed August 22, 2023).

[34] Bai J Perron P. Estimating and testing linear models with multiple structural changes. Econometrica. (1998) 66:47–78. 10.2307/2998540

[35] Bernal JL Cummins S Gasparrini A. Interrupted time series regression for the evaluation of public health interventions: a tutorial. Int J Epidemiol. (2017) 46(1):348–55. 10.1093/ije/dyw098 27283160 PMC5407170

[36] Wagner AK Soumerai SB Zhang F Ross-Degnan D. Segmented regression analysis of interrupted time series studies in medication use research. J Clin Pharm Ther. (2002) 27:299–309. 10.1046/j.1365-2710.2002.00430.x 12174032

[37] Ministry of Health and Long-Term Care. OHIP+: Children and youth pharmacare key facts for prescribers. (2023). Available at: https://www.afhto.ca/wp-content/uploads/ohip_prescriber-002.pdf (Accessed August 20, 2023).

[38] Cumby RE Huizinga J. Testing the autocorrelation structure of disturbances in ordinary least squares and instrumental variables regression. Econometrica. (1992) 60:185–95. 10.2307/2951684

[39] Newey WK West KD. A simple, positive semi-definite, heteroskedasticity and autocorrelation consistent covariance matrix. Econometrica. (1987) 55:703e8.

[40] Tsujimoto KC Cost KT LaForge-MacKenzie K Anagnostou E Birken CS Charach A School and learning contexts during the COVID-19 pandemic: implications for child and youth mental health. Curr Psychol. (2022):1–17. 10.1007/s12144-022-03941-y 36468159 PMC9685153

[41] Canadian Institute for Health Information. COVID-19’s impact on physician services. Available at: https://www.cihi.ca/en/covid-19-resources/impact-of-covid-19-on-canadas-health-care-systems/physician-services (Accessed August 22, 2023).

[42] Stewart SL Vasudeva AS Van Dyke JN Poss JW. Following the epidemic waves: child and youth mental health assessments in Ontario through multiple pandemic waves. Front Psychiatry. (2021) 12:730915. 10.3389/fpsyt.2021.730915 34867522 PMC8635704

[43] Courtney D Watson P Battaglia M Mulsant BH Szatmari P. COVID-19 impacts on child and youth anxiety and depression: challenges and opportunities. Can J Psychiatry. (2020) 65(10):688–91. 10.1177/0706743720935646 32567353 PMC7502880

[44] Asbury K Fox L Deniz E Code A Toseeb U. How is COVID-19 affecting the mental health of children with special educational needs and disabilities and their families? J Autism Dev Disord. (2021) 51(5):1772–80. 10.1007/s10803-020-04577-2 32737668 PMC7393330

[45] Deng J Zhou F Hou W Heybati K Lohit S Abbas U Prevalence of mental health symptoms in children and adolescents during the COVID-19 pandemic: a meta-analysis. Ann N Y Acad Sci. (2023) 1520:53–73. 10.1111/nyas.14947 36537131 PMC9880764

[46] Xie Y Xu E Al-Aly Z. Risks of mental health outcomes in people with COVID-19: cohort study. Br Med J. (2022) 376:e068993. 10.1136/bmj-2021-068993 35172971 PMC8847881

[47] Wade M Prime H Browne DT. Why we still need longitudinal mental health research with children and youth during (and after) the COVID-19 pandemic. Psychiatry Res. (2023) 323:115126. 10.1016/j.psychres.2023.115126 36989911 PMC9943557

[48] Bobo WV Cooper WO Stein CM Olfson M Graham D Daugherty J Antipsychotics and the risk of type 2 diabetes mellitus in children and youth. JAMA Psychiatry. (2013) 70:1067–75. 10.1001/jamapsychiatry.2013.2053 23965896

[49] Cohen D Bonnot O Bodeau N Consoli A Laurent C. Adverse effects of second-generation antipsychotics in children and adolescents: a bayesian meta-analysis. J Clin Psychopharmacol. (2012) 32(3):309–16. 10.1097/JCP.0b013e3182549259 22544019

[50] Pisano S Catone G Veltri S Lanzara V Pozzi M Clementi E Update on the safety of second generation antipsychotics in youths: a call for collaboration among paediatricians and child psychiatrists. Ital J Pediatr. (2016) 42:51. 10.1186/s13052-016-0259-2 27209326 PMC4875613

[51] Hsueh L Iturralde E Slama NE Spalding SR Sterling SA. Cardiometabolic monitoring and sociodemographic and clinical characteristics of youths prescribed antipsychotic medications. Psychiatr Serv. (2023) 74(8):801–8. 10.1176/appi.ps.20220151 37016828 PMC10539018

[52] Antoniou T Wang T Pajer K Gardner W Lunsky Y Penner M Adherence to antipsychotic laboratory monitoring guidelines in children and youth: a population-based study. Front Psychiatry. (2023) 14:1172559. 10.3389/fpsyt.2023.1172559 37252150 PMC10217777

[53] Chen W Cepoiu-Martin M Stang A Duncan D Symonds C Cooke L Antipsychotic prescribing and safety monitoring practices in children and youth: a population-based study in Alberta, Canada. Clin Drug Investig. (2018) 38:449–55. 10.1007/s40261-018-0626-4 29453686

[54] Antoniou T McCormack D Kitchen S Pajer K Gardner W Lunsky Y Geographic variation and sociodemographic correlates of prescription psychotropic drug use among children and youth in Ontario, Canada: a population-based study. BMC Public Health. (2023) 23:85. 10.1186/s12889-022-14677-6 36631810 PMC9832754

[55] Carnovale C Battini V Santoro C Riccio MP Carucci S Nobile M Review: association between antipsychotic drugs and metabolic syndrome hallmarks in children and adolescents. J Am Acad Child Adolesc Psychiatry. (2023):S0890-8567(23)00317-9. 37391174 10.1016/j.jaac.2023.04.018

