# Supplementary material for: Impact of the COVID-19 pandemic on antidepressant and antipsychotic use among children and adolescents: a population-based study
Source: Front Pediatr. 2023 Dec 11;11:1282845. doi: 10.3389/fped.2023.1282845 (PMC10749316; doi:10.3389/fped.2023.1282845)
Supplement: Supplementary file 1 [file Table1.docx]

**Timeline of Pandemic-Associated School Closures in Ontario, Canada**

| **Month** | **Timeline of School Closures and Reopenings** |
| --- | --- |
| 2020-03-14 | Schools closed |
| 2020-03-12 | First post-secondary school closure in province |
| 2020-09-08 | Public schools opened for 1st day of in-person learning; masks required for all staff and grade 4 to 12 students |
| 2021-01-04 | Public and private elementary and secondary school students moved to teacher-led remote learning (7 public health regions) |
| 2021-01-04 | Public and private secondary school students moved to teacher-led remote learning (27 public health regions) |
| 2021-01-12 | Mask use mandated indoors for grades 1 to 3, and outdoors for all students, with expanded testing and screening protocols |
| 2021-01-25 | Elementary/secondary schools resumed in-person learning with testing, asymptomatic screening and mask mandate for grades 1 to 3 (7 regions) |
| 2021-02-01 | Elementary and secondary schools resumed in-person learning (4 additional public health units) |
| 2021-02-08 | Elementary and secondary schools resumed in-person learning (13 additional public health units) |
| 2021-02-16 | Elementary and secondary schools resumed in-person learning in all 3 remaining public health units (Peel, Toronto and York Region) |
| 2021-04-19 | Public and private elementary and secondary schools moved to remote learning |
| 2021-09-07 | Schools open for the 2021–2022 school year; option of returning to in-person learning for full school day or synchronous remote learning |
| 2022-01-05 | All public and private school students resumed virtual learning, in-person return delayed |
| 2022-01-17 | Students returned to in-person learning |
| 2022-02-10 | Extra-curricular activities are allowed again in schools; students are required to wear a mask but may temporarily remove their mask for activities |

**Supplemental Table 1: Projected and Actual Antidepressant Dispensing in Children and Youth, April 2020 to March 2022 (Units per 1000 children and youth)**

| **Month** | **Projected Rate of Antidepressant dispensing in absence of COVID pandemic (units per 1000)** | **Actual Rate of Antidepressant dispensing during COVID pandemic (units per 1000)** | **Relative percent change, actual versus projected Antidepressant dispensing (95% confidence interval** |
| --- | --- | --- | --- |
| April 2020 | 812.8 | 622.1 | -23.5% (-23.6% to -23.3%) |
| May 2020 | 873.4 | 697.0 | -20.2% (-20.3% to -20.0%) |
| June 2020 | 876.3 | 945.1 | 7.8% (7.7% to 8.0%) |
| July 2020 | 828.2 | 968.8 | 20.7% (20.5% to 21.0%) |
| August 2020 | 853.7 | 800.0 | -6.3% (-6.5% to -6.1%) |
| September 2020 | 860.1 | 870.7 | 1.2% (1.0% to 1.4%) |
| October 2020 | 936.1 | 9753 | 4.2% (4.0% to 4.4%) |
| November 2020 | 955.8 | 959.6 | 0.39% (0.22% to 0.56%) |
| December 2020 | 983.5 | 1019.7 | 3.7% (3.5% to 3.9%) |
| January 2021 | 823.6 | 862.1 | 4.7% (4.5% to 4.9%) |
| February 2022 | 786.3 | 801.6 | 2.0% (1.8% to 2.1%) |
| March 2021 | 883.1 | 972.0 | 10.1% (9.9% to 10.3%) |
| April 2021 | 883.9 | 948.8 | 7.3% (7.2% to 7.5%) |
| May 2021 | 949.2 | 983.3 | 3.6% (3.4% to 3.8%) |
| June 2021 | 951.9 | 1011.4 | 6.3% (6.1% to 6.4%) |
| July 2021 | 899.1 | 992.8 | 10.4% (10.2% to 10.6%) |
| August 2021 | 926.3 | 1005.4 | 8.5% (8.4% to 8.7%) |
| September 2021 | 932.7 | 1012.0 | 8.5% (8.3% to 8.7%) |
| October 2021 | 1014.5 | 1074.5 | 5.9% (5.7% to 6.1%) |
| November 2021 | 1035.4 | 1160.6 | 12.1% (11.9% to 12.3%) |
| December 2021 | 1064.8 | 1194.2 | 12.2% (12.0% to 12.3%) |
| January 2022 | 891.2 | 979.1 | 9.9% (9.7% to 10.1%) |
| February 2022 | 850.4 | 920.0 | 8.2% (8.0% to 8.4%) |
| March 2022 | 954.6 | 1072.6 | 12.4% (12.2% to 12.5%) |

**Supplemental Table 2: Projected and Actual Antidepressant Dispensing in Children 0 to 13 years of age, April 2020 to March 2022**

| **Month** | **Projected Rate of Antidepressant dispensing in absence of COVID pandemic (units per 1000)** | **Actual Rate of Antidepressant dispensing during COVID pandemic (units per 1000)** | **Relative percent change, actual versus projected Antidepressant dispensing (95% confidence interval** |
| --- | --- | --- | --- |
| April 2020 | 239.1 | 199.6 | -16.5% (-16.9% to -16.2%) |
| May 2020 | 258.9 | 220.7 | -14.8% (-15.1% to -14.5%) |
| June 2020 | 262.1 | 287.5 | 9.7% (9.3% to 10.1%) |
| July 2020 | 263.0 | 291.9 | 11.0% (10.6% to 11.4%) |
| August 2020 | 262.4 | 258.8 | -1.4% (-1.7% to -1.0%) |
| September 2020 | 277.4 | 286.7 | 3.4% (3.0% to 3.7%) |
| October 2020 | 295.3 | 321.1 | 8.7% (8.4% to 9.1%) |
| November 2020 | 303.2 | 314.7 | 3.8% (3.5% to 4.2%) |
| December 2020 | 310.0 | 348.3 | 12.4% (12.0% to 12.7%) |
| January 2021 | 245.6 | 265.3 | 8.0% (7.6% to 8.4%) |
| February 2022 | 230.3 | 251.2 | 9.1% (8.7% to 9.5%) |
| March 2021 | 262.6 | 296.0 | 12.9% (12.5% to 13.3%) |
| April 2021 | 262.7 | 293.7 | 11.8% (11.4% to 12.2%) |
| May 2021 | 284.2 | 301.3 | 6.0% (5.6% to 6.4%) |
| June 2021 | 287.5 | 319.3 | 11.1% (10.7% to 11.4%) |
| July 2021 | 288.3 | 311.8 | 8.2% (7.8% to 8.5%) |
| August 2021 | 287.4 | 319.8 | 11.3% (10.9% to 11.7%) |
| September 2021 | 303.6 | 332.0 | 9.3% (9.0% to 9.7%) |
| October 2021 | 323.0 | 346.7 | 7.3% (7.0% to 7.7%) |
| November 2021 | 331.4 | 376.6 | 13.6% (13.3% to 14.0%) |
| December 2021 | 338.6 | 392.5 | 15.9% (15.5% to 16.3%) |
| January 2022 | 268.1 | 289.1 | 7.8% (7.4% to 8.2%) |
| February 2022 | 251.2 | 277.0 | 10.3% (9.9% to 10.7%) |
| March 2022 | 286.3 | 322.3 | 12.6% (12.2% to 12.9%) |

**Supplemental Table 3: Projected and Actual Antidepressant Dispensing in Children and Youth 14 to 18 years of age, April 2020 to March 2022**

| **Month** | **Projected Rate of Antidepressant dispensing in absence of COVID pandemic (units per 1000)** | **Actual Rate of Antidepressant dispensing during COVID pandemic (units per 1000)** | **Relative percent change, actual versus projected Antidepressant dispensing (95% confidence interval** |
| --- | --- | --- | --- |
| April 2020 | 2299.4 | 1720.9 | -25.2% (-25.3% to -25.0%) |
| May 2020 | 2465.7 | 1935.9 | -21.5% (-21.7% to -21.3%) |
| June 2020 | 2467.5 | 2655.5 | 7.6% (7.4% to 7.8%) |
| July 2020 | 2294.5 | 2729.3 | 19.0% (18.7% to 19.2%) |
| August 2020 | 2386.5 | 2207.5 | -7.5% (-7.7% to -7.3%) |
| September 2020 | 2370.9 | 2389.5 | 0.79% (.059% to 0.99%) |
| October 2020 | 2595.2 | 2676.7 | 3.1% (2.9% to 3.3%) |
| November 2020 | 2646.1 | 2636.7 | -0.35% (-0.54% to -0.17%) |
| December 2020 | 2726.5 | 2766.0 | 1.4% (1.3% to 1.6%) |
| January 2021 | 2325.9 | 2423.6 | 4.2% (4.0% to 4.4%) |
| February 2022 | 2230.7 | 2241.8 | 0.50% (0.29% to 0.71%) |
| March 2021 | 2494.9 | 2739.7 | 9.8% (9.6% to 10.0%) |
| April 2021 | 2497.0 | 2662.9 | 6.6% (6.4% to 6.8%) |
| May 2021 | 2676.0 | 2767.8 | 3.4% (3.2% to 3.6%) |
| June 2021 | 2676.5 | 2822.5 | 5.5% (5.3% to 5.7%) |
| July 2021 | 2487.4 | 2774.9 | 11.6% (11.3% to 11.8%) |
| August 2021 | 2585.8 | 2799.3 | 8.3% (8.1% to 8.5%) |
| September 2021 | 2567.5 | 2791.6 | 8.7% (8.5% to 8.9%) |
| October 2021 | 2808.9 | 2978.9 | 6.1% (5.9% to 6.2%) |
| November 2021 | 2862.5 | 3212.0 | 12.2% (12.0% to 12.4%) |
| December 2021 | 2948.1 | 3292.2 | 11.7% (11.5% to 11.9%) |
| January 2022 | 2513.6 | 2753.8 | 9.6% (9.4% to 9.8%) |
| February 2022 | 2409.5 | 2573.7 | 6.8% (6.6% to 7.0%) |
| March 2022 | 2693.6 | 3002.6 | 11.5% (11.3% to 11.7%) |

**Supplemental Table 4: Projected and Actual Antipsychotic Dispensing in Children and Youth, June 2020 to March 2022 (Units per 1000 children and youth)**

| **Month** | **Projected Rate of Antipsychotic dispensing in absence of COVID pandemic (units per 1000)** | **Actual Rate of Antipsychotic dispensing during COVID pandemic (units per 1000)** | **Relative percent change, actual versus projected Antipsychotic dispensing (95% confidence interval** |
| --- | --- | --- | --- |
| June 2020 | 266.1 | 307.4 | 15.5% (15.2% to 15.9%) |
| July 2020 | 264.3 | 299.1 | 13.2% (12.8% to 13.5%) |
| August 2020 | 260.4 | 274.0 | 5.2% (4.9% to 5.6%) |
| September 2020 | 260.8 | 274.5 | 5.2% (4.9% to 5.6%) |
| October 2020 | 275.2 | 302.8 | 10.0% (9.7% to 10.4%) |
| November 2020 | 275.5 | 298.7 | 8.4% (8.1% to 8.8%) |
| December 2020 | 279.7 | 319.1 | 14.1% (13.7% to 14.4%) |
| January 2021 | 253.8 | 269.8 | 6.3% (6.0% to 6.7%) |
| February 2022 | 236.4 | 256.1 | 8.3% (7.9% to 8.7%) |
| March 2021 | 259.6 | 295.1 | 13.7% (13.3% to 14.0%) |
| April 2021 | 251.5 | 286.4 | 13.9% (13.5% to 14.2%) |
| May 2021 | 262.9 | 292.4 | 11.2% (10.9% to 11.6%) |
| June 2021 | 264.9 | 299.9 | 13.2% (12.9% to 13.6%) |
| July 2021 | 263.1 | 302.6 | 15.0% (14.7% to 15.4%) |
| August 2021 | 259.2 | 310.8 | 19.9% (19.6% to 20.3%) |
| September 2021 | 259.6 | 305.2 | 17.6% (17.2% to 17.9%) |
| October 2021 | 274.0 | 301.9 | 10.2% (9.9% to 10.5%) |
| November 2021 | 274.3 | 308.0 | 12.3% (12.0% to 12.6%) |
| December 2021 | 278.5 | 319.2 | 14.6% (14.3% to 15.0%) |
| January 2022 | 252.5 | 282.0 | 11.7% (11.3% to 12.0%) |
| February 2022 | 235.2 | 255.3 | 8.6% (8.2% to 8.9%) |
| March 2022 | 258.4 | 286.7 | 11.0% (10.6% to 11.3%) |

**Supplemental Table 5: Projected and Actual Antipsychotic Dispensing in Children 0 to 13 years of age, June 2020 to March 2022**

| **Month** | **Projected Rate of Antipsychotic dispensing in absence of COVID pandemic (units per 1000)** | **Actual Rate of Antipsychotic dispensing during COVID pandemic (units per 1000)** | **Relative percent change, actual versus projected Antipsychotic dispensing (95% confidence interval** |
| --- | --- | --- | --- |
| June 2020 | 159.6 | 182.7 | 14.5% (13.9% to 15.0) |
| July 2020 | 157.4 | 183.5 | 16.6% (16.1% to 17.1%) |
| August 2020 | 156.8 | 173.3 | 10.5% (10.0% to 11.1%) |
| September 2020 | 159.3 | 169.4 | 6.4% (5.9% to 6.9%) |
| October 2020 | 169.2 | 192.3 | 13.6% (13.1% to 14.1%) |
| November 2020 | 170.1 | 189.1 | 11.2% (10.7% to 11.7%) |
| December 2020 | 169.8 | 197.6 | 16.4% (15.9% to 16.9%) |
| January 2021 | 146.1 | 160.5 | 9.8% (9.3% to 10.3%) |
| February 2022 | 136.1 | 148.6 | 9.2% (8.6% to 9.7%) |
| March 2021 | 151.4 | 175.1 | 15.7% (15.1% to 16.2%) |
| April 2021 | 146.1 | 169.4 | 15.9% (15.4% to 16.5%) |
| May 2021 | 151.5 | 173.3 | 14.4% (13.8% to 14.9%) |
| June 2021 | 156.0 | 173.8 | 11.4% (10.9% to 11.9%) |
| July 2021 | 153.8 | 178.9 | 16.3% (15.8% to 16.8%) |
| August 2021 | 153.2 | 181.8 | 18.7% (18.1% to 19.2%) |
| September 2021 | 155.7 | 180.4 | 15.8% (15.3% to 16.4%) |
| October 2021 | 165.7 | 180.1 | 8.7% (8.2% to 9.2%) |
| November 2021 | 166.5 | 183.7 | 10.3% (9.8% to 10.8%) |
| December 2021 | 166.2 | 190.4 | 14.5% (14.0% to 15.0%) |
| January 2022 | 142.6 | 160.0 | 12.2% (11.7% to 12.8%) |
| February 2022 | 132.6 | 144.3 | 8.8% (8.3% to 9.4%) |
| March 2022 | 147.8 | 165.6 | 12.0% (11.5% to 12.5%) |

**Supplemental Table 6: Projected and Actual Antipsychotic Dispensing in Children and Youth 14 to 18 years of age, June 2020 to March 2022**

| **Month** | **Projected Rate of Antipsychotic dispensing in absence of COVID pandemic (units per 1000)** | **Actual Rate of Antipsychotic dispensing during COVID pandemic (units per 1000)** | **Relative percent change, actual versus projected Antipsychotic dispensing (95% confidence interval** |
| --- | --- | --- | --- |
| June 2020 | 543.4 | 631.8 | 16.3% (15.8% to 16.7%) |
| July 2020 | 542.8 | 599.7 | 10.5% (10.1% to 10.9%) |
| August 2020 | 530.3 | 536.0 | 1.1% (0.65% to 1.5%) |
| September 2020 | 525.0 | 547.8 | 4.3% (3.9% to 4.8%) |
| October 2020 | 550.7 | 590.4 | 7.2% (6.8% to 7.6%) |
| November 2020 | 549.6 | 583.7 | 6.2% (5.8% to 6.6%) |
| December 2020 | 565.3 | 634.9 | 12.3% (11.9% to 12.8%) |
| January 2021 | 535.4 | 556.0 | 3.9% (3.4% to 4.3%) |
| February 2022 | 499.2 | 537.3 | 7.6% (7.2% to 8.1%) |
| March 2021 | 542.6 | 609.0 | 12.2% (11.8% to 12.7%) |
| April 2021 | 527.1 | 592.6 | 12.4% (12.0% to 12.9%) |
| May 2021 | 553.9 | 604.1 | 9.1% (8.6% to 9.5%) |
| June 2021 | 549.3 | 629.8 | 14.6% (14.2% to 15.1%) |
| July 2021 | 548.7 | 626.5 | 14.2% (13.7% to 14.6%) |
| August 2021 | 536.2 | 648.3 | 20.9% (20.4% to 21.4%) |
| September 2021 | 530.9 | 631.9 | 19.0% (18.5% to 19.5%) |
| October 2021 | 556.6 | 620.6 | 11.5% (11.0% to 11.9%) |
| November 2021 | 555.6 | 633.3 | 14.0% (13.5% to 14.4%) |
| December 2021 | 571.3 | 656.3 | 14.9% (14.4% to 15.3%) |
| January 2022 | 541.3 | 595.6 | 10.0% (9.6% to 10.5%) |
| February 2022 | 505.1 | 541.0 | 7.1% (6.7% to 7.6%) |
| March 2022 | 548.5 | 598.3 | 9.1% (8.6% to 9.5%) |
| April 2022 | 543.4 | 631.8 | 16.3% (15.8% to 16.7%) |
| May 2022 | 542.8 | 599.7 | 10.5% (10.1% to 10.9%) |
| June 2022 | 530.3 | 536.0 | 1.1% (0.65% to 1.5%) |
